# Exploiting the Genomic Diversity of Rice (*Oryza sativa* L.): SNP-Typing in 11 Early-Backcross Introgression-Breeding Populations

**DOI:** 10.3389/fpls.2018.00849

**Published:** 2018-06-22

**Authors:** Jauhar Ali, Umair M. Aslam, Rida Tariq, Varunseelan Murugaiyan, Patrick S. Schnable, Delin Li, Corinne M. Marfori-Nazarea, Jose E. Hernandez, Muhammad Arif, Jianlong Xu, Zhikang Li

**Affiliations:** ^1^International Rice Research Institute, Los Baños, Philippines; ^2^Institute of Crop Science, University of the Philippines Los Baños, Los Baños, Philippines; ^3^National Institute of Biotechnology and Genetic Engineering, Faisalabad, Pakistan; ^4^Data2Bio, LLC, Ames, IA, United States; ^5^Department of Agronomy, Iowa State University, Ames, IA, United States; ^6^Department of Plant Genetics and Breeding, China Agricultural University, Beijing, China; ^7^National Key Facility for Crop Gene Resources and Genetic Improvement, Institute of Crop Science, Chinese Academy of Agricultural Sciences, Beijing, China; ^8^Agricultural Genomics Institute at Shenzhen, Chinese Academy of Agricultural Sciences, Shenzhen, China

**Keywords:** SNP-typing, tunable genotyping by sequencing (tGBS), conventional genotyping by sequencing (cGBS), introgression breeding, non-synonymous SNPs, marker-assisted breeding

## Abstract

This study demonstrates genotyping-by-sequencing-based single-nucleotide polymorphism (SNP)-typing in 11 early-backcross introgression populations of rice (at BC_1_F_5_), comprising a set of 564 diverse introgression lines and 12 parents. Sequencing using 10 Ion Proton runs generated a total of ∼943.4 million raw reads, out of which ∼881.6 million reads remained after trimming for low-quality bases. After alignment, 794,297 polymorphic SNPs were identified, and filtering resulted in LMD50 SNPs (low missing data, with each SNP, genotyped in at least 50% of the samples) for each sub-population. Every data point was supported by actual sequencing data without any imputation, eliminating imputation-induced errors in SNP calling. Genotyping substantiated the impacts of novel breeding strategy revealing: (a) the donor introgression patterns in ILs were characteristic with variable introgression frequency in different genomic regions, attributed mainly to stringent selection under abiotic stress and (b) considerably lower heterozygosity was observed in ILs. Functional annotation revealed 426 non-synonymous deleterious SNPs present in 102 loci with a range of 1–4 SNPs per locus and 120 novel SNPs. SNP-typing this diversity panel will further assist in the development of markers supporting genomic applications in molecular breeding programs.

## Introduction

Rice is considered as one of the world’s most important staple foods and is the key to food security especially under the threats of climate change in the coming decades. Currently, rice is planted in 166 million hectares worldwide, nurturing some four billion people, and is harvested annually with a total worth of USD 203 billion (Global Rice Science Partnership [GRiSP], 2013).

The predicament caused by climate change and a burgeoning population is leading to increased food insecurity and poverty. It is imperative to hasten the rate of genetic improvement efforts to meet the challenges of these biophysical and socio-natural constraints. Breeding improved rice cultivars using cutting-edge biotechnological tools and delivering them efficiently within shorter time frames is the fundamental solution to this.

Cost-effective next-generation sequencing has been successfully employed for whole genome sequencing, gene expression, and single-nucleotide polymorphism (SNP) discovery (Xu et al., 2011; Harper et al., 2012; Li et al., 2014; The 3,000 Rice Genomes Project, 2014). Several approaches and methods are already developed for SNP discovery and genotyping in several crop species (Elshire et al., 2011; Wang et al., 2012).

Genotyping-by-sequencing (GBS) has also emerged as a powerful breeding tool with the continued increase of sequencing output, the development of reference genomes, and improved bioinformatics. Plant scientists are getting deep into connecting phenotype to genotype using sequencing outputs. Deciphering interactions among heritable genetic factors and phenotypes will aid in harnessing the benefits of genomics-assisted selection in plant breeding. GBS has the potential to discover novel or population-specific polymorphisms. One approach to using GBS is to incorporate polymorphisms discovered via GBS into a closed platform, like an array, which can then be used to genotype an entire population of interest (Poland and Rife, 2012). These array-based, large-scale SNP discovery pipelines have advanced considerably in the identification of chromosome-specific SNPs involved in stress tolerance mechanisms (Akpinar et al., 2017; Lucas et al., 2017).

Conventional GBS (cGBS; Elshire et al., 2011; Poland and Rife, 2012; Poland et al., 2012; Li et al., 2015) involves multiplexing samples using DNA barcoded adapters and a reduction in genome complexity (using, for example, restriction endonucleases to target only a small portion of the genome) to produce high-quality polymorphism data at a relatively much lower cost per sample. This approach has been demonstrated to be robust across a range of species and to be capable of producing enormous amounts of molecular markers (Elshire et al., 2011; Poland et al., 2012; Li et al., 2015).

However, cGBS comes with some complexities such as yielding relatively fewer reads per site and having high levels of missing data across samples. Low read depths per site limit the ability of cGBS to identify heterozygous loci in diversity panels while high levels of missing data require imputation that limits the ability of cGBS to detect rare alleles.

Like many modified and improved versions of cGBS, the tunable genotyping-by-sequencing (tGBS^®^) technology (Schnable et al., 2013) overcomes these two challenges of cGBS by amplifying and, therefore, sequencing fewer sites. This results in a given number of sequence reads being distributed across fewer sites of the genome, thereby yielding more reads per site. This higher read depth per site results in less missing data from an individual to individual, which provides more repeatability and enhances the usability of the resulting genotyping data. Also, because of this higher read depth, it is possible to accurately call heterozygous loci and confidently detect novel or rare alleles.

There are two basic modifications in tGBS^®^ relative to cGBS^®^. First, tGBS uses barcoded single-stranded oligos instead of the double-stranded adapters used in cGBS, thereby eliminating the problem of the inter-molecular ligation of adapter molecules, making the entire process simpler, and improving the quality of results. Second, the modification that overcomes the read depth problem associated with cGBS involves a selective amplification of restriction fragments. The amount of genome reduction levels (GRLs) can be tuned. For example, in GRL1, GRL2, and GRL3, only 1/4^th^, 1/16^th^, and 1/64^th^ of all of the restriction fragments are sequenced. To achieve these levels of genome reduction, about one to three additional nucleotides are added up at the end of a selective polymerase chain reaction (PCR) primer. These GRLs concentrate the available sequencing reads at fewer sites of the genome, thereby increasing read depth and enhancing the accuracy of SNP calling, including in heterozygous individuals, as well as the discovery of rare alleles (Schnable, 2013; Schnable et al., 2013; Islam et al., 2015).

In this study, the application of tGBS^®^ for SNP-typing 11 rice populations, comprising a diverse set of 564 introgression lines (ILs) and the corresponding 12 parents, is extensively described. It is the first report on the application of tGBS^®^ in rice and its application for SNP-typing. Another nested goal of this study is to serve as a positional reference for chromosomal introgressions to the ongoing molecular breeding work of the authors. Subsequent publications based on the detailed analysis of the tGBS^®^ results briefly summarized here will follow describing the fine mapping of various abiotic and biotic stress tolerance loci, their transcriptome and metabolome analyses, and their utilization in our marker-assisted breeding programs.

## Materials and Methods

Eleven donors (elite cultivars selected from different rice agro-ecologies) were crossed with Weed Tolerant Rice-1 (WTR-1), and their F_1_s were backcrossed once also with WTR-1 (**Table 1**). Subsequently, the F_1_BC_1_s were self-pollinated, and their seeds were bulked to create 11 BC_1_F_2_ populations that were screened for three rounds beginning in the 2011 wet season under varied biotic and abiotic stresses such as drought, low input, salinity, submergence, tungro, and standard irrigated conditions, among others.

**Table 1:** SNP summary and parent genotype categories’ ratios.

Sub-pop^∗^	Donor parent (DP) and source country	No. of ILs	LMD50 SNPs
1	Haoannong (DP1) – China	120	4,669
2	Cheng-Hui 448 (DP4) – China	67	5,968
3	Feng-Ai-Zan (DP5) – China	42	2,284
4	Y 134 (DP6) – China	34	4,035
5	Zhong 413 (DP7) – China	56	3,149
6	Khazar (DP8) – Iran	29	3,962
7	BG 300 (DP9) – Sri Lanka	60	5,226
8	OM 997 (DP10) – Vietnam	55	5,921
9	Basmati-385 (DP12) – Pakistan	31	5,995
10	M 401 (DP17) – United States	33	7,045
11	X 21 (DP19) – Vietnam	37	6,985
Total	11 DPs	564	55,239

*^∗^All 11 sub-populations have a common recipient parent, Weed Tolerant Rice-1 (WTR-1).*

**FIGURE 1:**
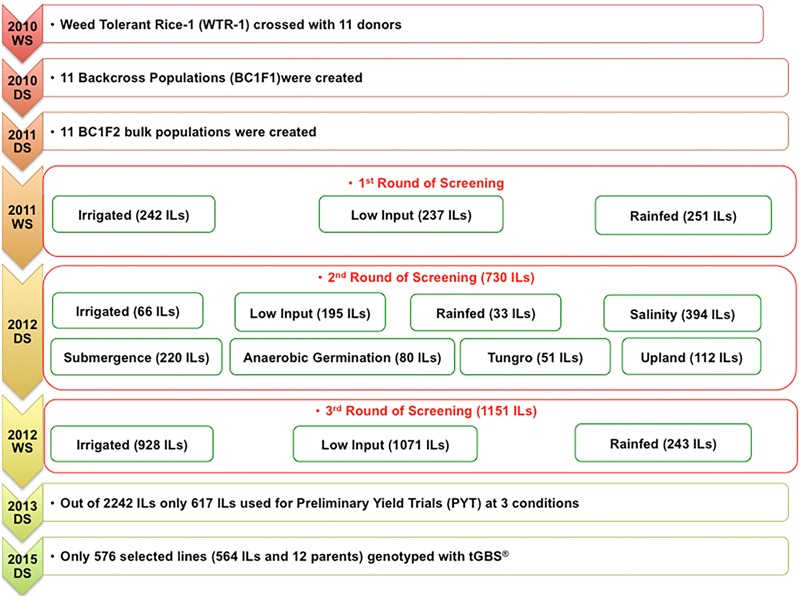
Illustrated breeding scheme.

In all environments, surviving plants that exhibited superior performance over the checks and the WTR-1 recipient parent (RP) were selected using a previously published approach as illustrated briefly in **Figure 1**. This novel early-backcross breeding technique has been proven successful in exploiting favorable genes hidden in diverse germplasms to develop ILs that are tolerant to multiple stresses (Ali et al., 2006, 2012a,b, 2013).

This process yielded 564 ILs derived from the 11 BC_1_F_5_ sub-populations, each of which was derived from one of the 11 different donor parents (DPs). These 564 ILs, 11 DPs, and the common RP were genotyped via tGBS^®^ (**Table 1** and **Supplementary Figure S11**). All the lines were grown in a greenhouse at the International Rice Research Institute (IRRI), Los Baños, Philippines, in 2015. The harvested leaves were stored at -80°C before extracting the genomic DNA using QIAGEN^®^ DNeasy Plant Mini Kits following QIAGEN^®^’s standard protocol (DNeasy^®^ Plant Handbook DNeasy Plant Mini Kit and tissues, or fungi Sample & Assay Technologies QIAGEN Sample and Assay Technologies, 2015) (QIAGEN, 2015). DNA quantification was performed using a Nanodrop 2000 spectrophotometer (Thermo Fisher Scientific, Waltham, MA, United States) as well as via agarose (1.5%) gel electrophoresis. One microgram of DNA per sample with a minimum concentration of 10 ng/μl and a 260/280 ratio of 1.7–2.0 was lyophilized and sent to D2B for tGBS^®^ analysis.

### tGBS^®^ Pipeline

All 576 samples were sequenced using 10 Ion Proton runs. The rice reference genome (Osativa_204_v7.0.fa) was downloaded from the Phytozome website^1^ [Rice (*Oryza sativa*) Reference Genome on Phytozome]. The typical workflow of tGBS^®^ analysis with a reference genome is illustrated in **Supplementary Figure S1**.

Library preparation and sequencing was performed using the previously published protocol by Data2Bio (11). In short, all DNA samples were digested using two restriction enzymes (*Nsp*I and *Bfu*CI). Ligation followed with a single-stranded barcode oligonucleotide in one site and an oligonucleotide complementary to amplification primer in the other site (Islam et al., 2015).

Trimming of raw sequence reads was conducted using Lucy software (Chou et al., 1998; Li and Chou, 2004). Nucleotides at each site were scanned for low quality and the bases with a PHRED quality value of <15, i.e., error rates of ≤3%, were removed (Ewing and Green, 1998). Trimmed reads were aligned to the public reference genome using GSNAP (Wu and Nacu, 2010; Islam et al., 2015). Confidently mapped reads were used for SNP discovery (≤2 mismatches for every 36 bp and <5 bases for every 75 bp as unaligned tails). Polymorphisms at each potential SNP site were carefully examined (supported by at least three reads) and putative homozygous and heterozygous SNPs were identified. Homozygous SNPs were called following the criteria of having a PHRED base quality of 20 (≤1% error rate) and at least three reads supporting the major common allele. Heterozygous SNPs were called if there were at least two reads supporting each of at least two different alleles, and further each of the two read types separately comprised >20% of the reads aligning to that site, and also if the sum of the reads supporting those two alleles comprised at least 90% of all reads covering the site. SNP calls were then filtered by a missing data rate of ≤80% having an allele number of 2, a number of genotypes of ≥2, minor allele frequency of ≥0.1, and heterozygosity range of 0–10% (Islam et al., 2015; Leiboff et al., 2015). Finally, the LMD50 (low missing data) SNP dataset was defined by filtering again by a missing data rate of ≤50%. The complete tGBS genotypic data have been archived at IRRI Dataverse^2^.

### Genomic Distribution and Annotation Analysis of Identified SNPs (LMD50)

Circos diagrams were generated using J-Circos to visualize the genomic distributions of the identified LMD50 SNPs for each sub-population on all chromosomes (An et al., 2015). Phylogenetic analyses based on the LMD50 SNPs were performed using Dissimilarity Analysis and Representation for Windows (DARwin v.6.0.013) to generate a neighbor-joining tree (Perrier et al., 2003; Perrier and Jacquemoud-Collet, 2006).

The structural and functional annotation of the identified LMD50 SNP dataset was performed using SNiPlay (Dereeper et al., 2015) and the latest version of rice genome assembly from the Rice Genome Annotation Project database (Kawahara, 2013). SNPs within coding regions were classified as either synonymous or non-synonymous. Non-synonymous LMD50 SNPs were further classified as tolerated/neutral or deleterious based on the change in amino acid sequence and its predicted impact on protein function using SIFT 4G (Vaser et al., 2016). An amino acid substitution was classified as deleterious if its SIFT score was ≤0.05 and tolerated or neutral if its SIFT score was >0.05. Among non-synonymous SNPs, the large-effect SNPs were separated and their functions determined using the MSU7 rice reference genome (Kawahara, 2013). J-Circos was used to visualize the distribution of SNPs with predicted deleterious effects on protein function within biotic and abiotic-related loci (An et al., 2015). These deleterious SNPs were individually plotted based on their physical positions (bp) on the 12 rice chromosomes for each of the 11 sub-populations. SNPs present in gene regions were identified based on their genomic position coordinates as provided in the MSU7 rice reference genome (Kawahara, 2013).

## Results and Discussion

The tGBS^®^ analysis was used for SNP-typing a rice diversity panel comprising 12 parents and 564 ILs. The use of multiple parents in the development of ILs through backcrosses is advantageous in genetic mapping as it harnesses more allelic diversity, thereby enabling the detection of more promising QTLs with more precision as compared to using bi-parental populations (Zhu et al., 2015; Ali et al., 2017).

Previously, tGBS^®^ has been shown to have several advantages. In a study involving upland cotton (*Gossypium hirsutum* L.; Islam et al., 2015), the tGBS^®^ protocol (Schnable et al., 2013) yielded more high-quality SNPs with higher read depths per SNP site than cGBS (Elshire et al., 2011).

The 943.4 M raw tGBS^®^ sequencing reads used in the current study were generated using 10 Ion Proton runs. After trimming low-quality bases, 881.6 M reads, and 87.8% of base pairs were retained. Approximately 80.9% and 65.7% of the trimmed reads could be aligned non-uniquely and uniquely, respectively. Using the reads from the 576 samples that uniquely aligned to the reference genome, 794,297 polymorphic sites were identified after interrogating 2,679,180 bases that have ≥5 reads in at least 50% of the samples.

**FIGURE 2:**
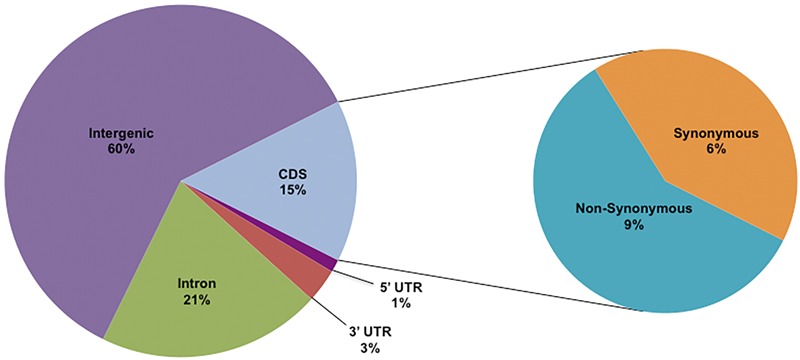
Annotation of LMD50 SNPs. Intergenic and genic proportions of identified SNPs.

After filtering (see section “Materials and Methods”), a low-missing dataset (LMD50) was identified (**Table 1**). Of the total LMD50 SNPs discovered in all 11 sub-populations (**Table 1**), ∼40% were located in genic regions (**Figure 2**). This frequency of SNPs in the coding region is higher than that reported in earlier studies (Arai-Kichise, 2011; Subbaiyan, 2012; Jain et al., 2014; Mehra et al., 2015); 4% of SNPs were detected in regulatory regions. Exonic or CDS regions contained 4,784 non-synonymous SNPs and 3,373 synonymous SNPs. As expected, the majority of SNPs (21%) located in the genic region were intronic.

**FIGURE 3:**
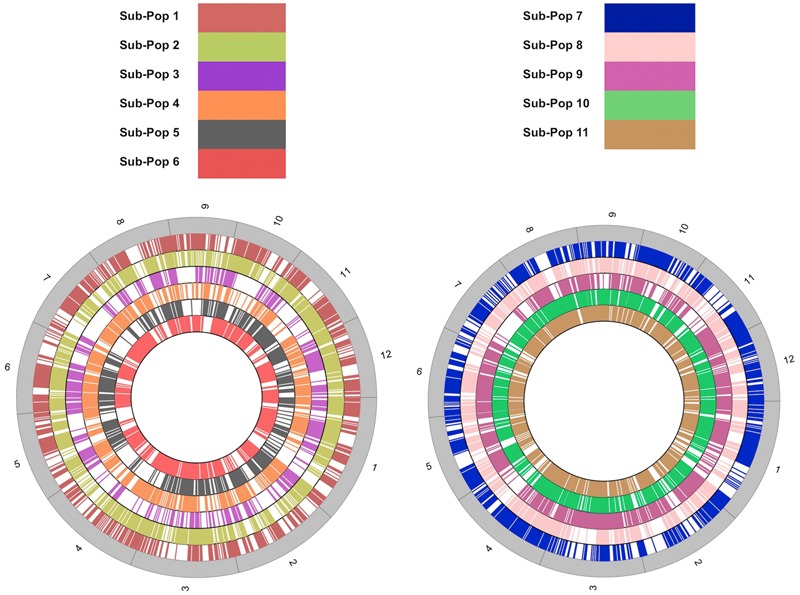
Genomic distribution of LMD50 SNPs. The size of each chromosome is based on actual chromosome lengths from the Rice Genome Annotation Project (MSU7; http://rice.plantbiology.msu.edu/index.shtml) database.

The LMD50 SNPs were not uniformly distributed across chromosomes (**Figure 3**). SNP distribution varied within chromosomes for all the 11 sub-populations. The analysis revealed major SNP hotspots across all chromosomes and genomic regions where no SNPs were identified. The variation in the distribution of polymorphisms on chromosomal basis has frequently been reported in rice and model plants (Feltus et al., 2004; Nordborg et al., 2005; Arai-Kichise, 2011; Hu, 2014). This localized effect of the chromosomal distribution of SNPs is attributed to GBS technologies which rely on uniquely aligned reads resulting in a non-uniform distribution of unique sequences (Elshire et al., 2011; Schnable et al., 2013) and natural selection-sweeps during rice domestication (Caicedo, 2007).

Sub-population 1 consisted of 122 samples (120 ILs and the two parents, **Table 1**). Using the unique alignments of each read from the 122 samples relative to the public reference genome, 4,669 high-quality SNPs (LMD50) were identified (**Table 1**).

**FIGURE 4:**
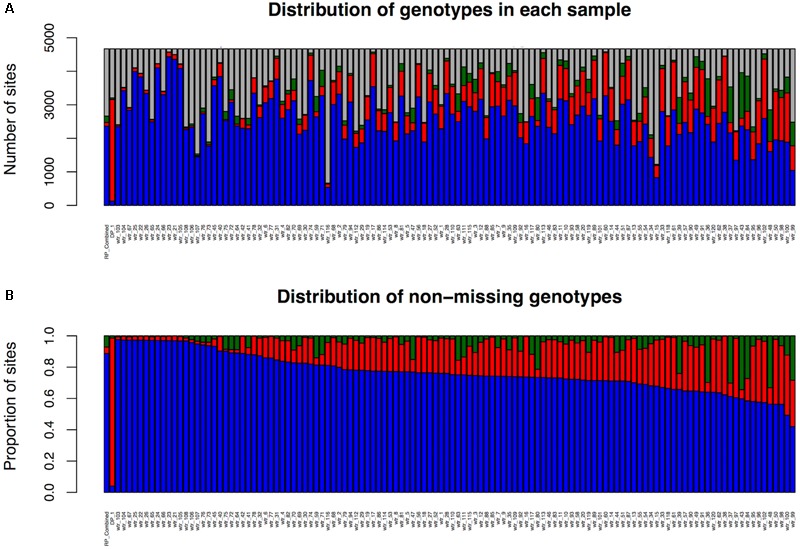
LMD50 SNPs’ genotype summary by sample for sub-population 1. **(A)** Including missing SNP calls. **(B)** Considering only non-missing SNP calls. Red color represents homozygous donor parent alleles, blue represents homozygous recurrent parent alleles, and green for heterozygous.

The number of LMD50 SNPs per sample that are homozygous for the recurrent parent allele, homozygous for the DP allele, and heterozygous and missing is shown in **Figure 4**, which also illustrates the proportion of the SNPs per sample that are homozygous for the recurrent parent allele, homozygous for the donor allele, or heterozygous among non-missing genotypes. Key figures for the LMD50 SNPs for the other 10 sub-populations are provided in **Supplementary Figures S2–S4**.

The LMD50 SNPs for each sample in sub-population 1 were plotted by their physical ordering on concatenated chromosomes, providing a clear visualization of genotypic patterns (**Supplementary Figure S5**). This display of LMD50 SNPs allows the reader to more easily observe clustering of SNPs that are heterozygous or homozygous for the DP allele in specific chromosomal regions. It is notable that the introgression patterns do not appear to be random, providing suggestive evidence of selection during backcrossing and in subsequent inbreeding generations. Most of the 11 sub-populations exhibited similar introgression patterns, noticeably corresponding to the breeding strategy for their development. Similar displays of LMD50 SNPs for the other 10 sub-populations are provided in **Supplementary Figures S6–S8**.

It is notable that on chromosome 4, considerable evidence of selection sweep exists, implying the increased frequency of donor alleles in response to stringent selection pressure under abiotic stress. A more in-depth study is required, and an effort is under progress to investigate the presumed selective sweeps in contrast with the standing variation where a selected variant predates the selection pressure (Peter et al., 2012). Similar occurrences of selective sweeps are evident on various chromosomes in other introgression populations and are indexed in **Supplementary Figures S6–S8**.

The recurrent parent allele frequency was also plotted by SNPs and selective window scanning (window size: 10 SNPs and step size: 5 SNPs were used; **Supplementary Figure S9**). Additionally, to visualize the unexpected high DP allele frequency presumably implying to selective sweep on chromosome 4 in comparison with chromosome 10, the same window scanning parameters were used and average DP allele frequency of each window was plotted (**Supplementary Figure S9**).

Recurrent parent allele frequency for each population by SNPs and selective window scanning (a window size of 10 SNPs and a step size of 5 SNPs were used) were plotted and aggregated for a broader visualization. The average recurrent parent allele frequency ranged between 0.7 and 0.8 for those populations (**Supplementary Figures S10, S11**).

**FIGURE 5:**
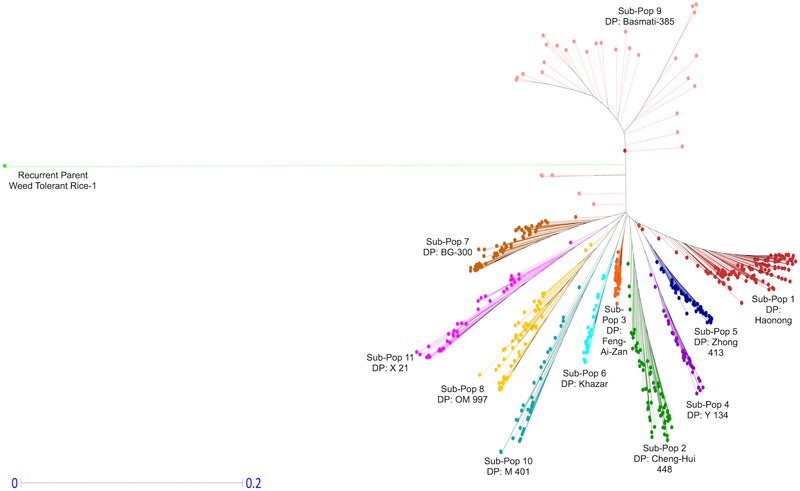
Diversity and phylogenetic patterns among 11 sub-populations. Neighbor-joining tree based on LMD50 SNPs.

The phylogenetic analysis based on the LMD50 SNPs revealed a clear differentiation of the 576 genotypes into 12 distinct groups in a neighbor-joining tree (**Figure 5**). The 12 groups correspond to the 11 DPs of each of the 11 sub-populations (**Table 1**) and the RP. The genetic distance between the RP and the 564 ILs can be explained by the novel breeding strategy used in this study (Ali et al., 2006, 2012a,b, 2013, 2017). Each IL arising from the cross between each of the 11 DPs and a single RP underwent rigorous screening and selection under various abiotic stresses. The whole panel of 564 genotypes possesses varying levels of tolerance toward these multiple stresses in the field screenings (unpublished data). The introgression event of single backcrossing and subsequent stringent screening and selection plausibly resulted in major functional introgressions from the donor genomes and quick fixation of the consequent genetic effects through novel breeding strategy. The evident genomic introgression in each IL from the DP (**Supplementary Figures S5–S8**) is consistent with the hypothesis that the introgressed genomic regions contributed to the strong phenotypic responses in resulting ILs, rendering them tolerant to multiple biotic and abiotic stresses during stringent selection. The favorable phenotypic effects get fixed in the population through positive selection leading to uniqueness and deviance from the RP, both genotypically and phenotypically.

Among the 4,784 non-synonymous SNPs, 426 were predicted based on SIFT analysis to confer deleterious effect on gene function and were predicted to be highly detrimental, having a tolerance index of 0.00. In all the 11 sub-populations, 102 loci contained 120 deleterious large-effect SNPs, with 1–4 SNPs per loci.

**FIGURE 6:**
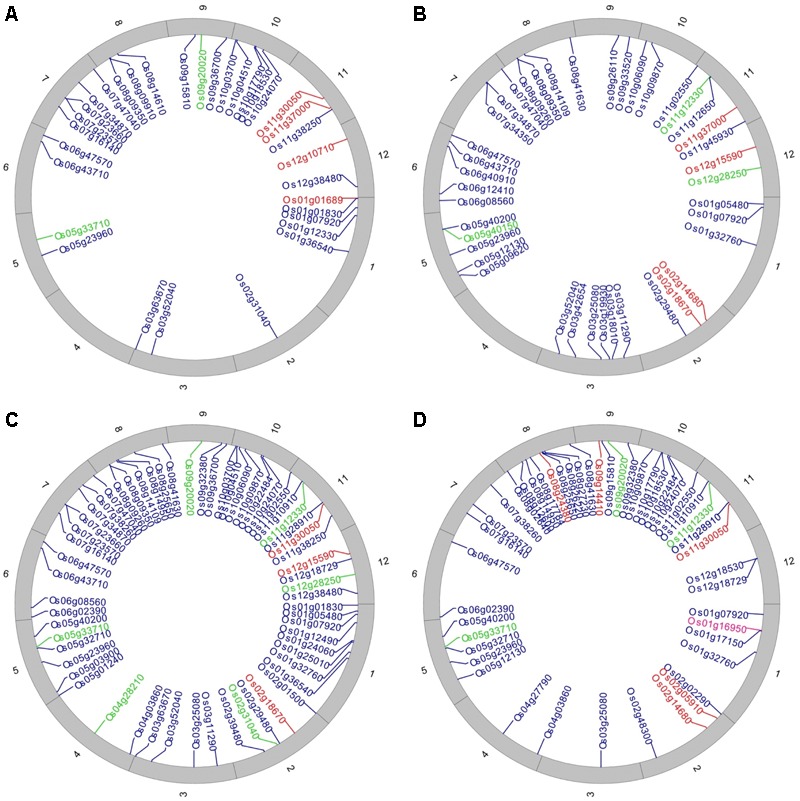
Distribution of non-synonymous SNPs that have a deleterious effect on proteins. Circos diagrams representing annotated gene locations in **(A)** sub-population 1 (*DP: Haoannong*), **(B)** sub-population 9 (*DP: Basmati-385*), **(C)** sub-population 10 (*DP: M-401*), and **(D)** sub-population 11 (*DP: X-21*). The outer numbering shows the 12 rice chromosomes. Red color shows the abiotic stress-related loci, green color shows biotic stress-related loci, and blue color shows the loci other than stress-related families.

Of the 102 affected loci, 24 were predicted to be responsive to biotic (six loci) and abiotic (18 loci) stress. These loci contained deleterious SNPs that substitute the amino acid and change the function of protein either positively or negatively. The identified abiotic and biotic stress-responsive loci are represented in Circos diagrams (**Figure 6** and **Supplementary Figures S12, S13**), and the corresponding details of amino acid and functional change are found in **Supplementary Tables S1, S2**.

In sub-populations 1 (*Dp: Haoannong*) and 4 (*DP: Y-134*), a G/A deleterious SNP at position 349,360 in the Os01g01689 locus alters an amino acid from *Ala* to *Thr* (**Supplementary Tables S1, S2**). This locus is associated with the abiotic stimulus. The DP *Haoannong* of sub-population one is known for its drought and salinity tolerance (Li and Xu, 2007) while the *Y-134* donor is good for agronomic traits – characteristics that were used as the basis for their selection as DPs in the early-backcross introgression-breeding program. A large-effect deleterious allele was observed in sub-populations 9 (*DP: Basmati-385*), 10 (*DP: M-401*), and 11 (*DP: X-21*) at position 6,884,254 in the Os11g12330 locus and the changed codon A/C changes the amino acid from *Lys* to *Asn* (**Supplementary Tables S1, S2**). The presence of this SNP was observed in the DPs while there was no variation in the RP at this position, suggesting that this allele was introgressed from the DPs in the sub-populations. High variability was observed at position 16,687,362 of the Os12g28250 locus within sub-populations 7 (*DP: BG-300*), 9 (*DP: Basmati-385*), and 10 (*DP: M-401*) as these three DPs have “G” at this position, the same as in the reference genome (MSU7), while the RP has SNP “C” (**Supplementary Tables S1, S2**). The presence of this allele precisely defines the background of the RP (WTR-1) in the sub-populations (**Supplementary Datasheet S2**).

Through the novel early-backcross introgression-breeding strategy used in this study, many important tolerance alleles were combined due to selections made simultaneously in different stress conditions over three rounds using these sub-populations (**Figure 1**).

## Conclusion

This study discussed the utility of tGBS^®^ in rice for SNP-typing 11 early-backcross introgression populations. Genotyping substantiated the impacts of novel breeding strategy revealing: (a) the donor introgression patterns in ILs were characteristic with variable introgression frequency in different genomic regions, attributed mainly to stringent selection under abiotic stress and (b) considerably lower heterozygosity was observed in ILs. The development of SNP markers through further detailed analysis of the sequencing results summarized here will help in the identification of novel gene and QTL resources for biotic and abiotic stress tolerance in rice for use in marker-assisted breeding programs.

## Author Contributions

JA bred the genotypic population and conceived the research. JA, UA, and CM-N prepared the DNA samples and sent for genotyping. PS, DL, UA, RT, JA, and VM analyzed the data and prepared the results. All authors prepared, read, revised, and approved the manuscript.

## Conflict of Interest Statement

PS and DL are employed by company Data2Bio, LLC, Ames, IA, United States, where we carried out the tunable genotyping by sequencing for our materials. Both read the MS and placed their inputs. The remaining authors declare that the research was conducted in the absence of any commercial or financial relationships that could be construed as a potential conflict of interest.

## Footnotes

1 http://phytozome.jgi.doe.gov/pz/portal.html#!info?alias=Org_Osativa

2 https://doi.org/10.7910/DVN/RRXCR3

## Supplementary Material

FIGURE S1 tGBS workflow.

FIGURE S2 LMD50 SNPs’ genotype summary by sample for **(a)** sub-population 2, **(b)** sub-population 3, **(c)** sub-population 4, and **(d)** sub-population 5.

FIGURE S3 LMD50 SNPs’ genotype summary by sample for **(a)** sub-population 6, **(b)** sub-population 7, **(c)** sub-population 8, and **(d)** sub-population 9.

FIGURE S4 LMD50 SNPs’ genotype summary by sample for **(a)** sub-population 10 and **(b)** sub-population 11.

FIGURE S5 Chromosomal representation of SNPs (LMD50) for sub-population 1. Red color indicates donor parent allele, blue color represents recurrent parent allele, and green color represents heterozygous loci.

FIGURE S6 LMD50 SNPs’ representation on chromosomal basis for **(a)** sub-population 2, **(b)** sub-population 3, **(c)** sub-population 4, and **(d)** sub-population 5.

FIGURE S7 LMD50 SNPs representation on chromosomal basis for **(a)** sub-population 6, **(b)** sub-population 7, **(c)** sub-population 8, and **(d)** sub-population 9.

FIGURE S8 LMD50 SNPs representation on chromosomal basis for **(a)** sub-population 10 and **(b)** sub-population 11.

FIGURE S9 Allele frequencies in sub-population 1. **(a)** Recurrent parent allele frequency plotted by SNPs (LMD50) among 12 chromosomes. **(b)** Recurrent parent allele frequency plotted by window scanning method (a window size of 10 SNPs and a step size of 5 SNPs were used). **(c)** Donor parent allele frequency on chromosome 4 [a window size of 10 SNPs and a step size of 5 SNPs were used; the black line (0.25) represents average donor parent allele frequency among all population’s parental LMD50 SNPs]. **(d)** Donor parent allele frequency on chromosome 10 [a window size of 10 SNPs and a step size of 5 SNPs were used; the black line (0.25) represents average donor parent allele frequency among all population’s parental LMD50 SNPs].

FIGURE S10 Recurrent parent allele frequencies of introgression populations by SNPs (LMD50). Frequency distribution of 12 chromosomes represented for each population titled with its donor parent (DP) identity.

FIGURE S11 Recurrent parent allele frequencies of introgression populations by window scanning method. Frequency distribution of 12 chromosomes represented for each population titled with its donor parent (DP) identity. A window size of 10 SNPs and a step size of 5 SNPs were used.

FIGURE S12 Distribution of non-synonymous deleterious SNPs for **(a)** sub-population 2, **(b)** sub-population 3, **(c)** sub-population 4, and **(d)** sub-population 5.

FIGURE S13 Distribution of non-synonymous deleterious SNPs for **(a)** sub-population 6, **(b)** sub-population 7, and **(c)** sub-population 8.

TABLE S1 Functional details of identified deleterious SNPs in biotic and abiotic loci.

TABLE S2 Functional details of identified non-synonymous deleterious SNPs excluding biotic and abiotic loci.

DATASHEET S2 List of genotypes used for tGBS along with coding.
