# Supplementary figures and images for: Exploiting the Genomic Diversity of Rice (Oryza sativa L.): SNP-Typing in 11 Early-Backcross Introgression-Breeding Populations
Source: Front Plant Sci. 2018 Jun 22;9:849. doi: 10.3389/fpls.2018.00849 (PMC6024854; doi:10.3389/fpls.2018.00849)

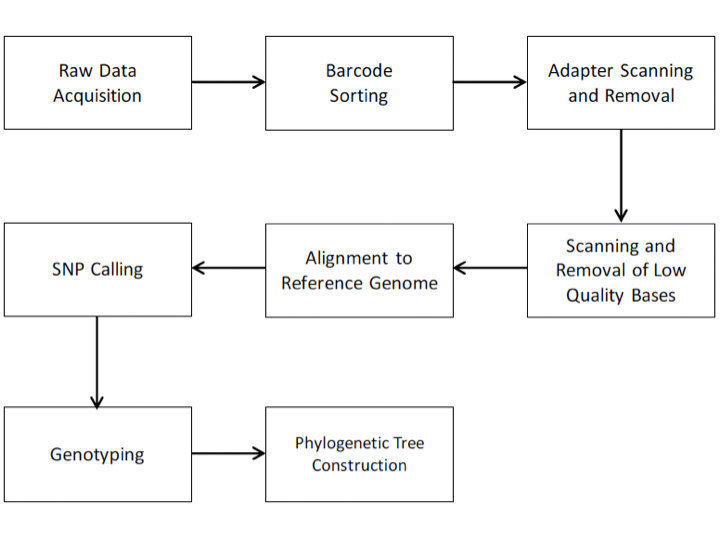

Supplement: FIGURE S1 — tGBS workflow. [file Image_1.JPEG]

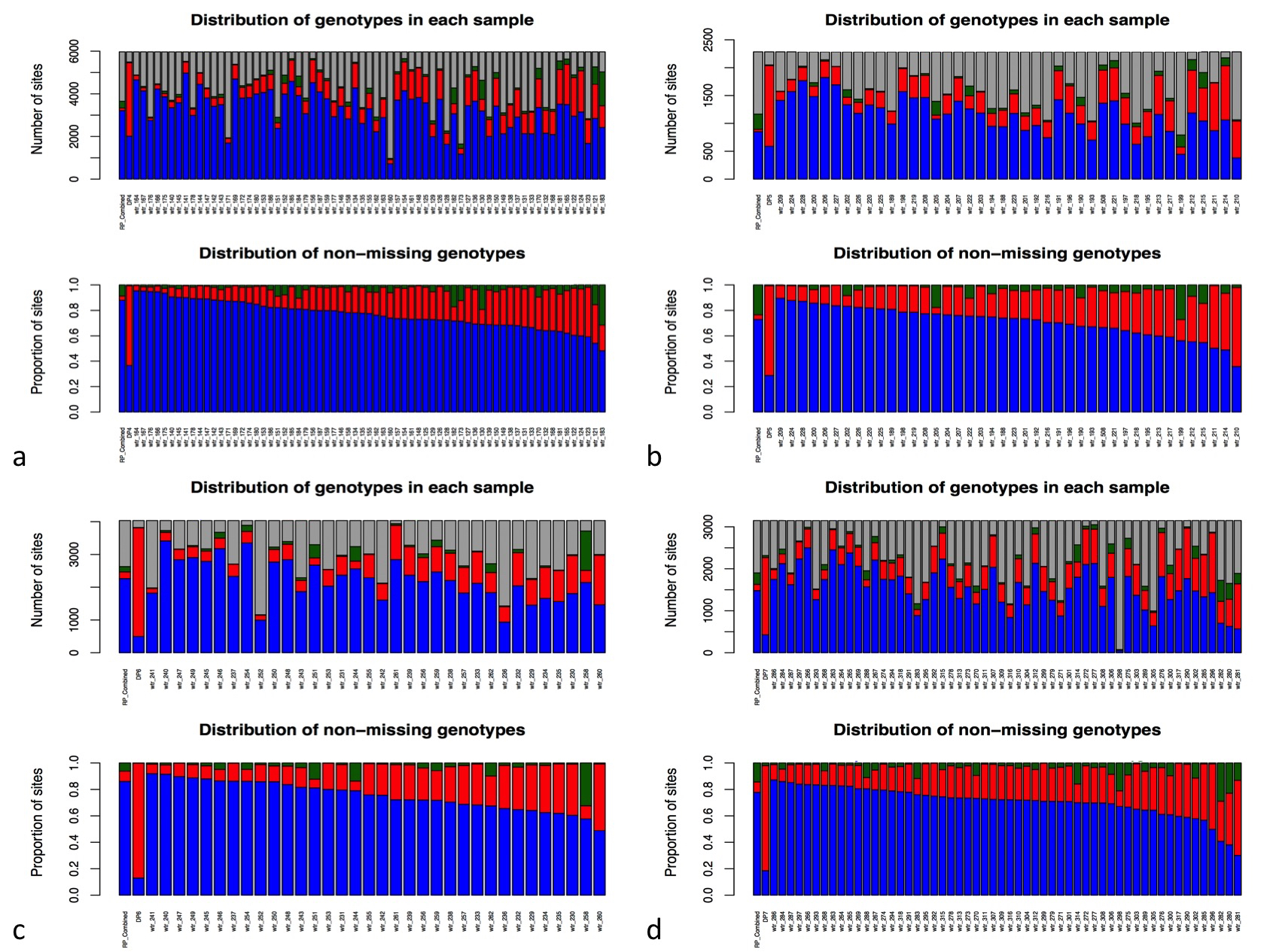

Supplement: FIGURE S2 — LMD50 SNPs’ genotype summary by sample for (a) sub-population 2, (b) sub-population 3, (c) sub-population 4, and (d) sub-population 5. [file Image_2.JPEG]

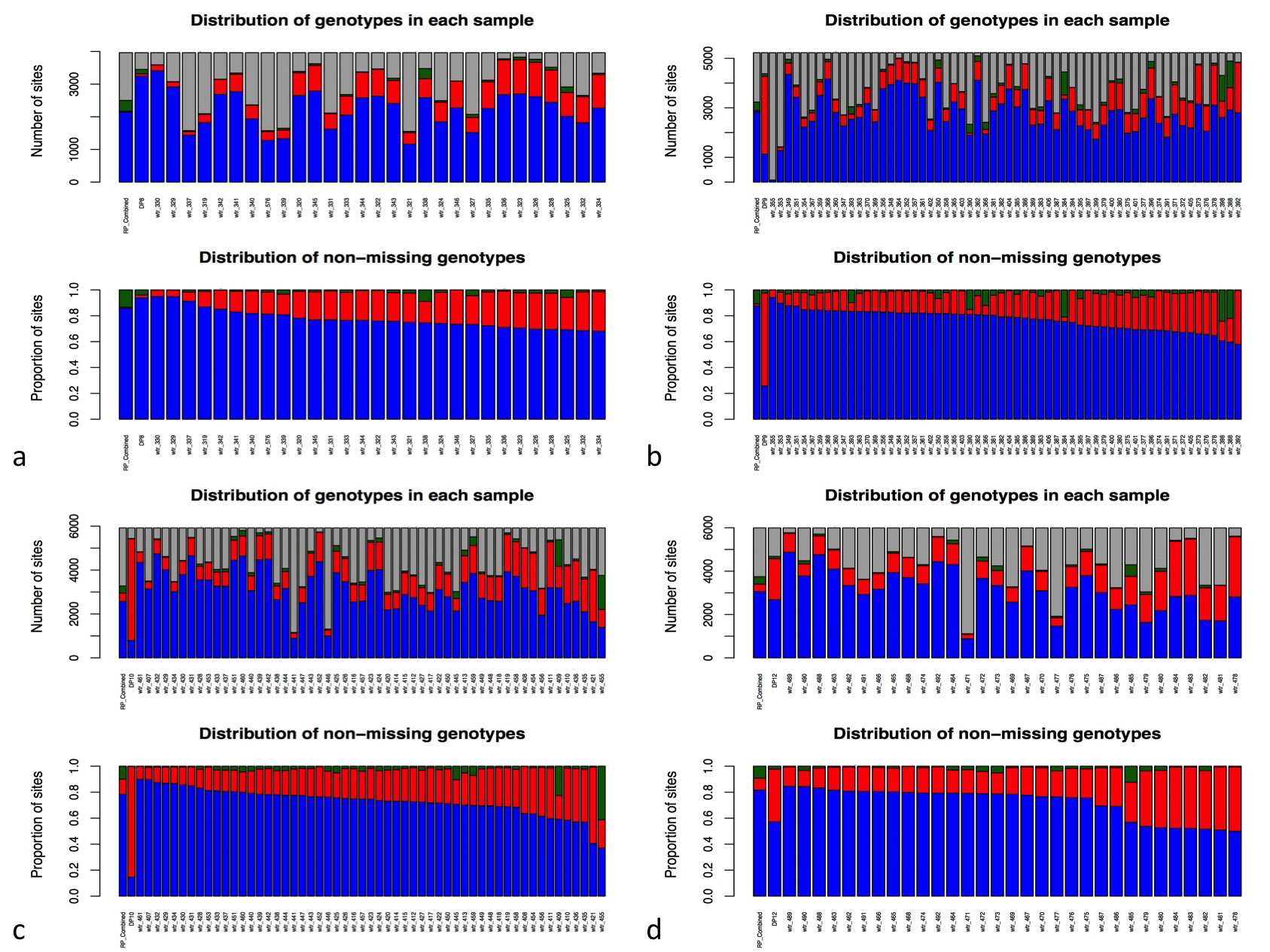

Supplement: FIGURE S3 — LMD50 SNPs’ genotype summary by sample for (a) sub-population 6, (b) sub-population 7, (c) sub-population 8, and (d) sub-population 9. [file Image_3.JPEG]

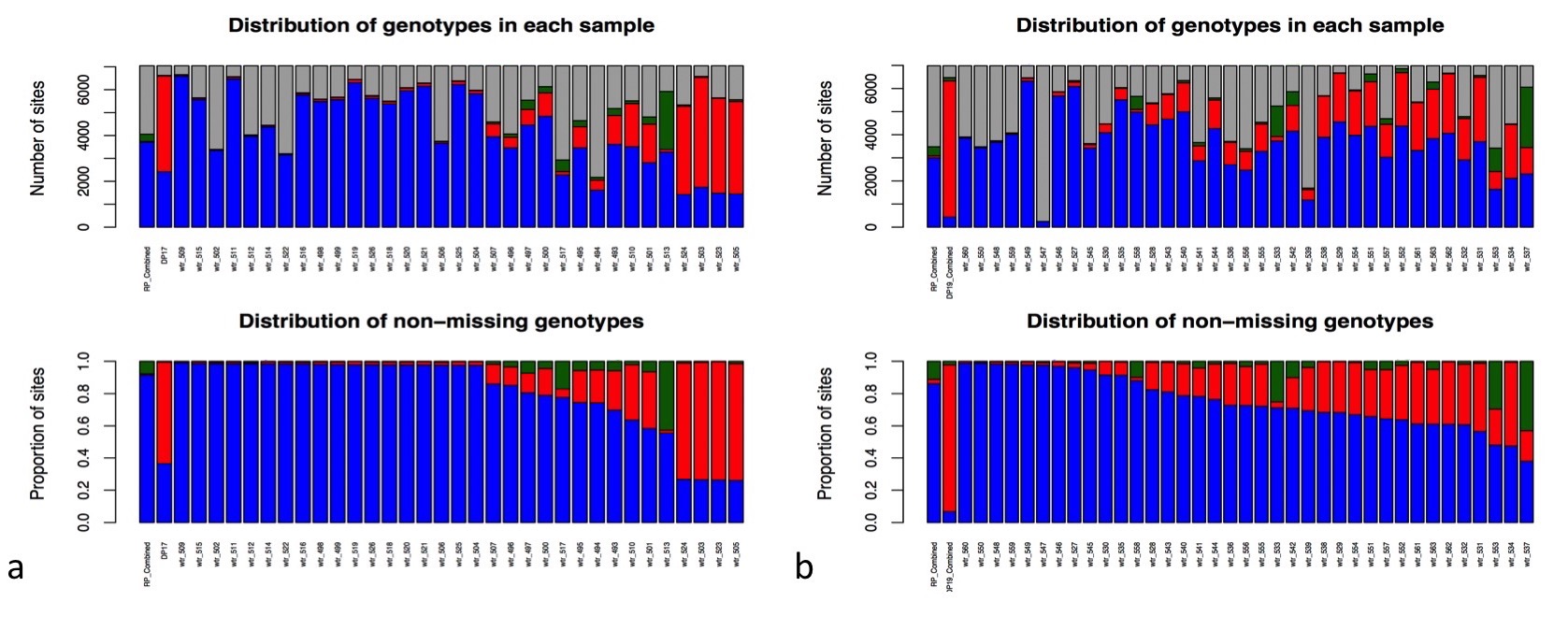

Supplement: FIGURE S4 — LMD50 SNPs’ genotype summary by sample for (a) sub-population 10 and (b) sub-population 11. [file Image_4.JPEG]

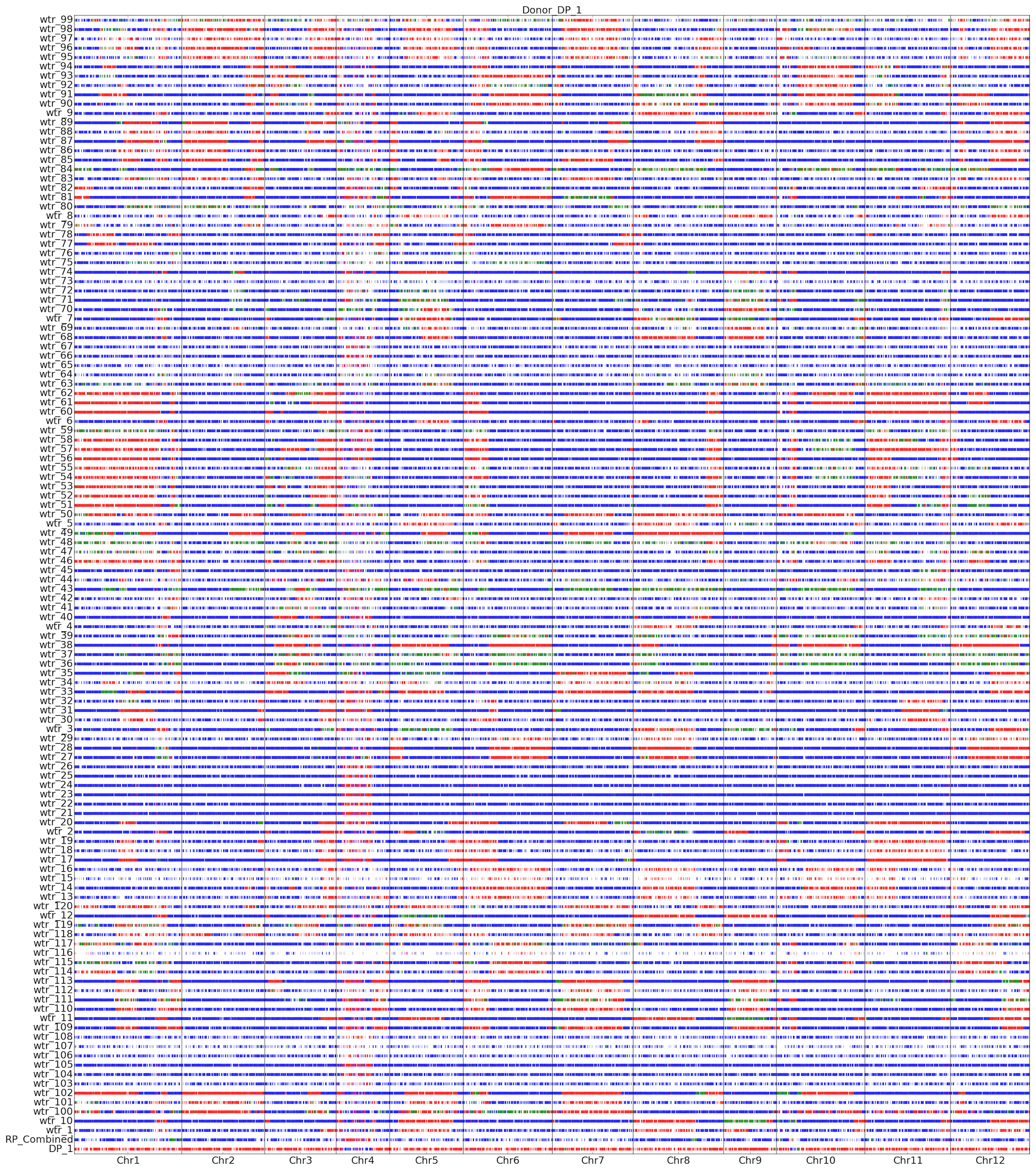

Supplement: FIGURE S5 — Chromosomal representation of SNPs (LMD50) for sub-population 1. Red color indicates donor parent allele, blue color represents recurrent parent allele, and green color represents heterozygous loci. [file Image_5.JPEG]

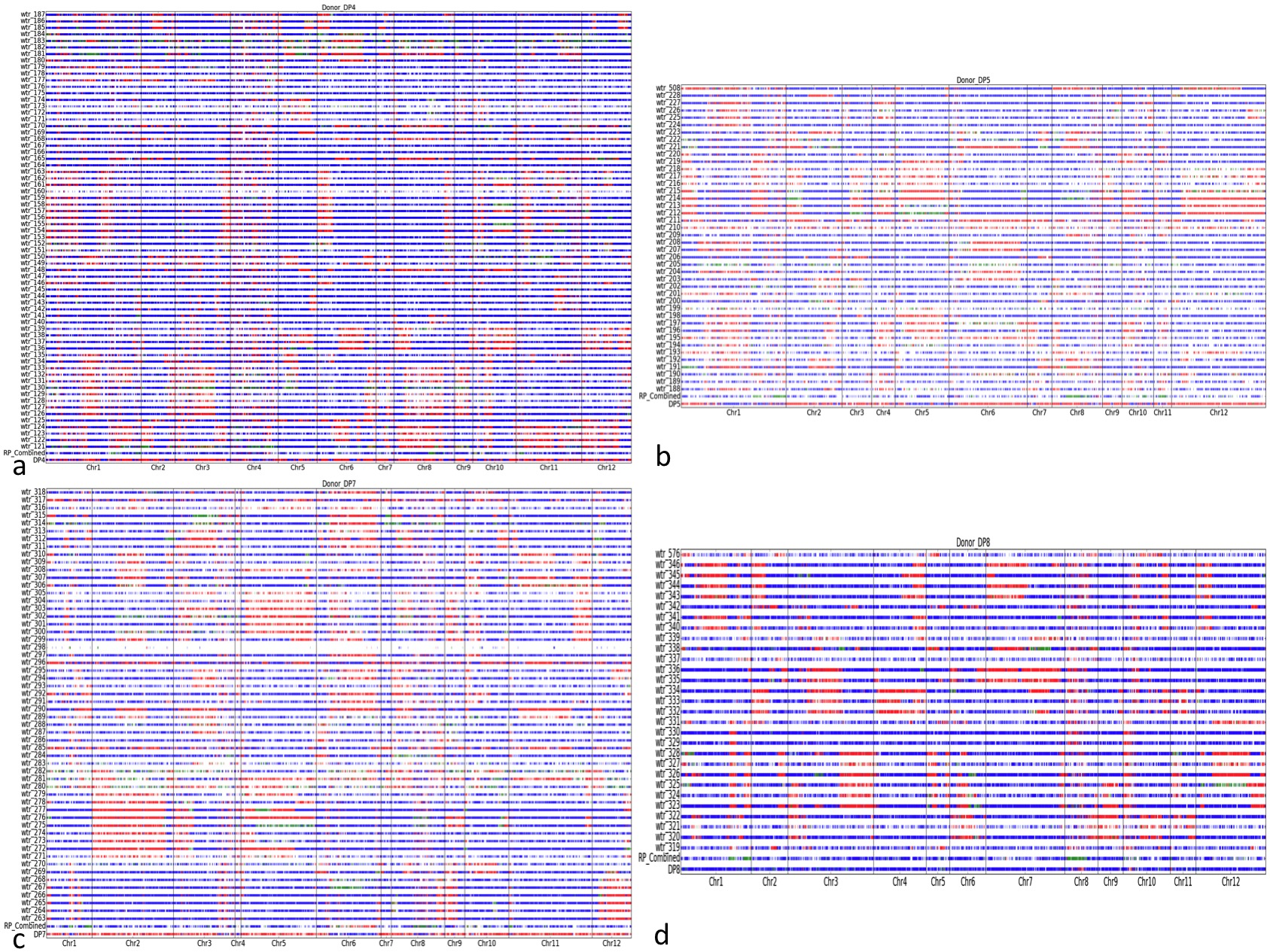

Supplement: FIGURE S6 — LMD50 SNPs’ representation on chromosomal basis for (a) sub-population 2, (b) sub-population 3, (c) sub-population 4, and (d) sub-population 5. [file Image_6.JPEG]

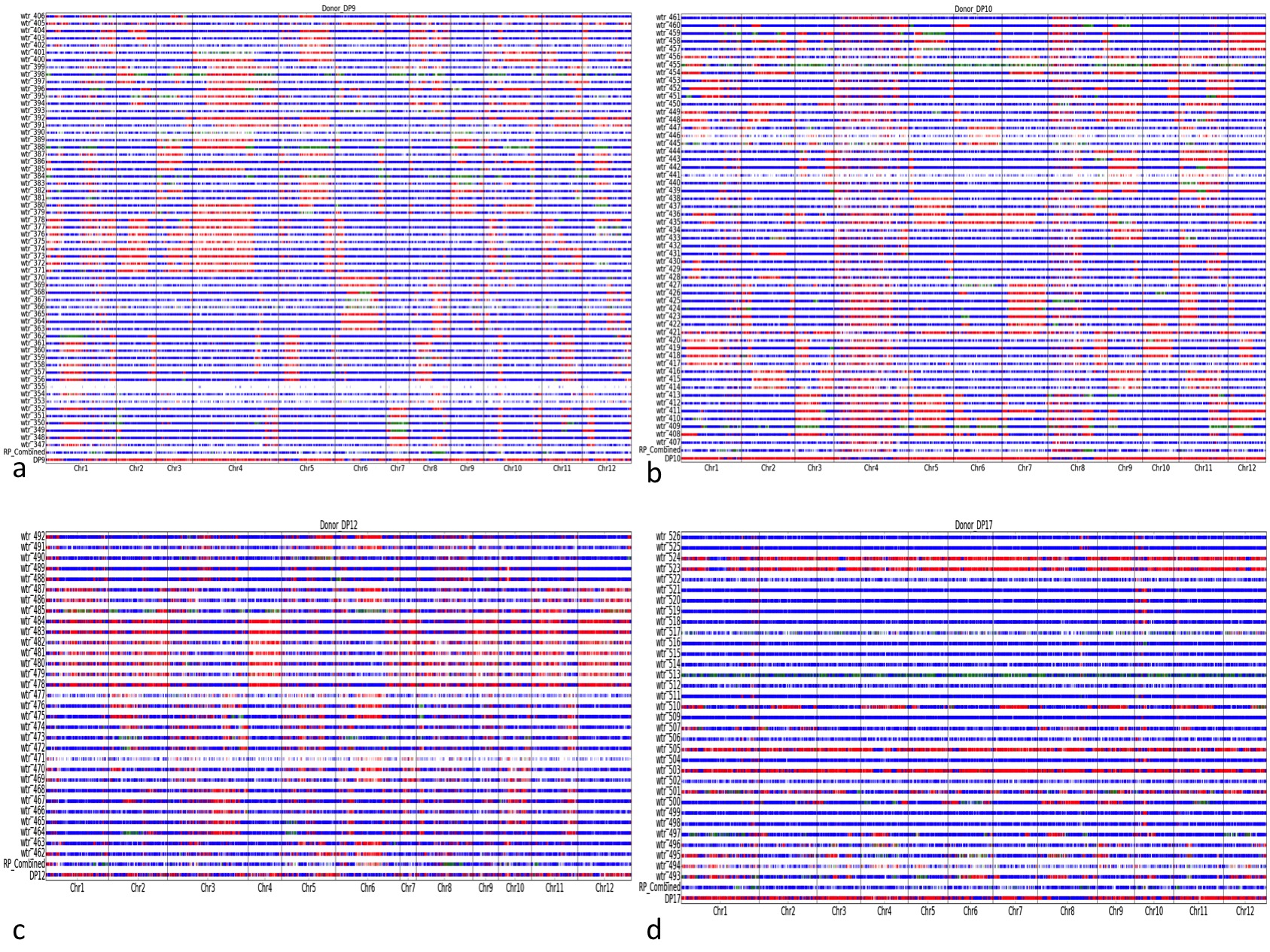

Supplement: FIGURE S7 — LMD50 SNPs representation on chromosomal basis for (a) sub-population 6, (b) sub-population 7, (c) sub-population 8, and (d) sub-population 9. [file Image_7.JPEG]

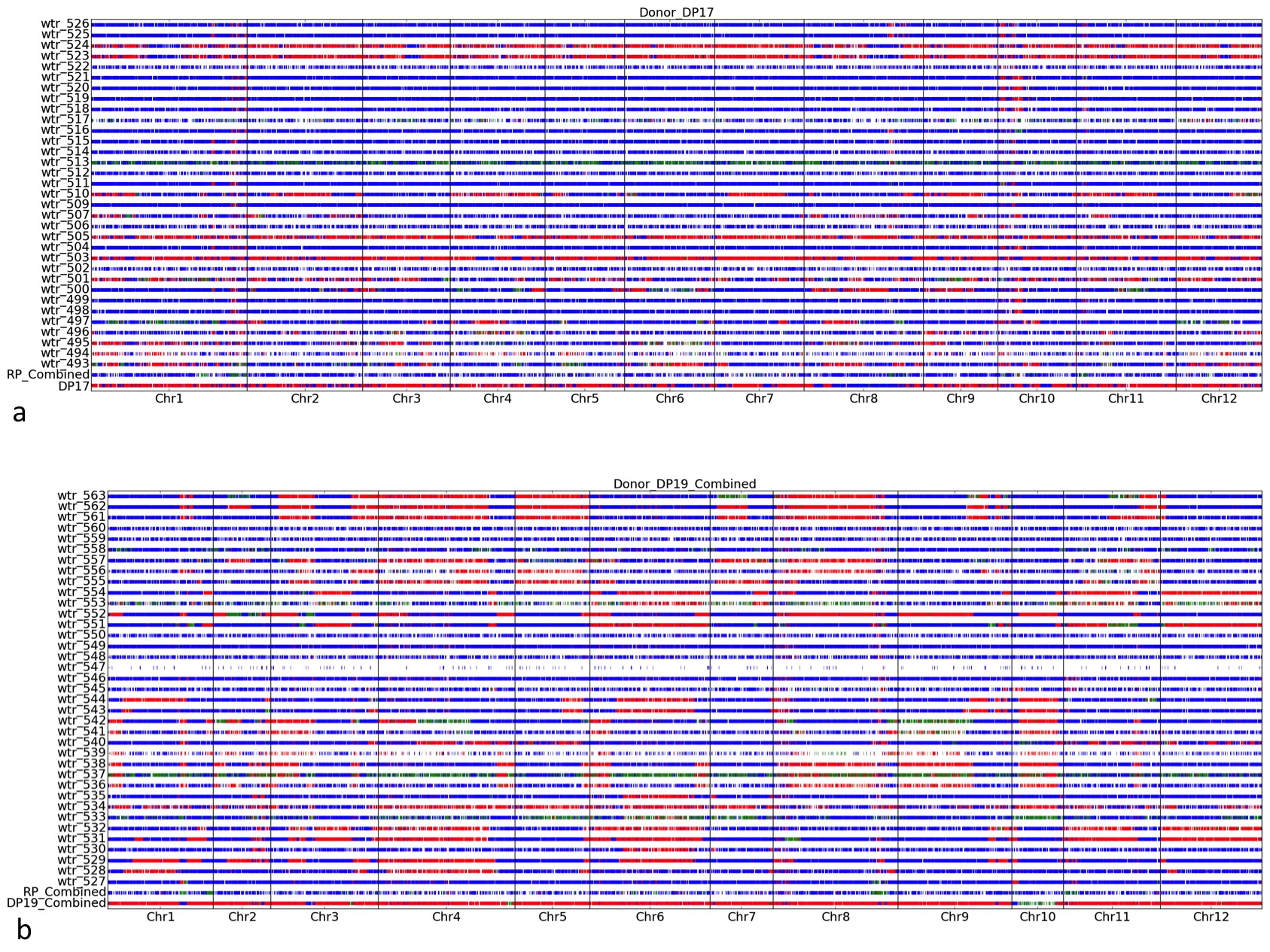

Supplement: FIGURE S8 — LMD50 SNPs representation on chromosomal basis for (a) sub-population 10 and (b) sub-population 11. [file Image_8.JPEG]

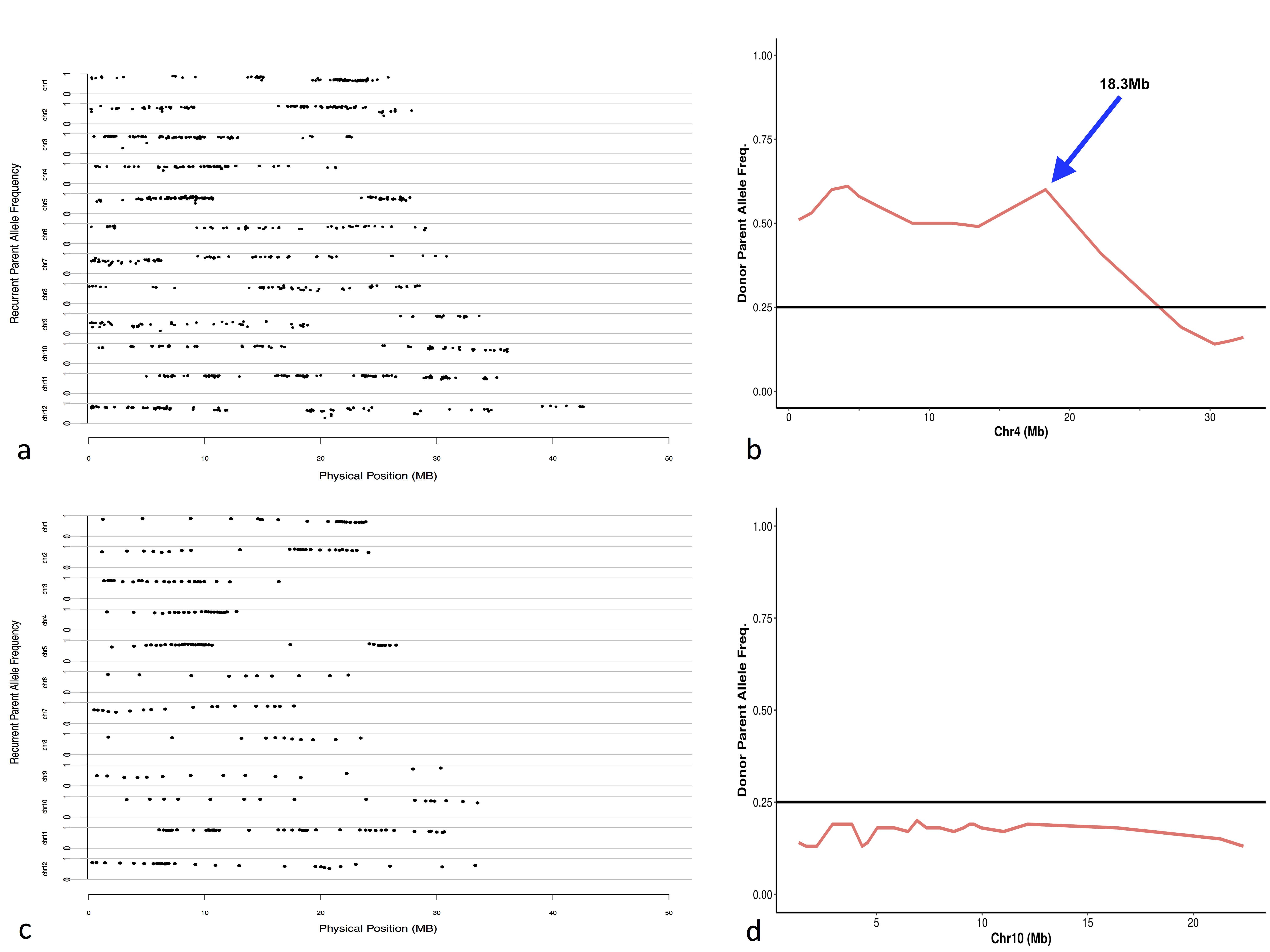

Supplement: FIGURE S9 — Allele frequencies in sub-population 1. (a) Recurrent parent allele frequency plotted by SNPs (LMD50) among 12 chromosomes. (b) Recurrent parent allele frequency plotted by window scanning method (a window size of 10 SNPs and a step size of 5 SNPs were used). (c) Donor parent allele frequency on chromosome 4 [a window size of 10 SNPs and a step size of 5 SNPs were used; the black line (0.25) represents average donor parent allele frequency among all population’s parental LMD50 SNPs]. (d) Donor parent allele frequency on chromosome 10 [a window size of 10 SNPs and a step size of 5 SNPs were used; the black line (0.25) represents average donor parent allele frequency among all population’s parental LMD50 SNPs]. [file Image_9.JPEG]

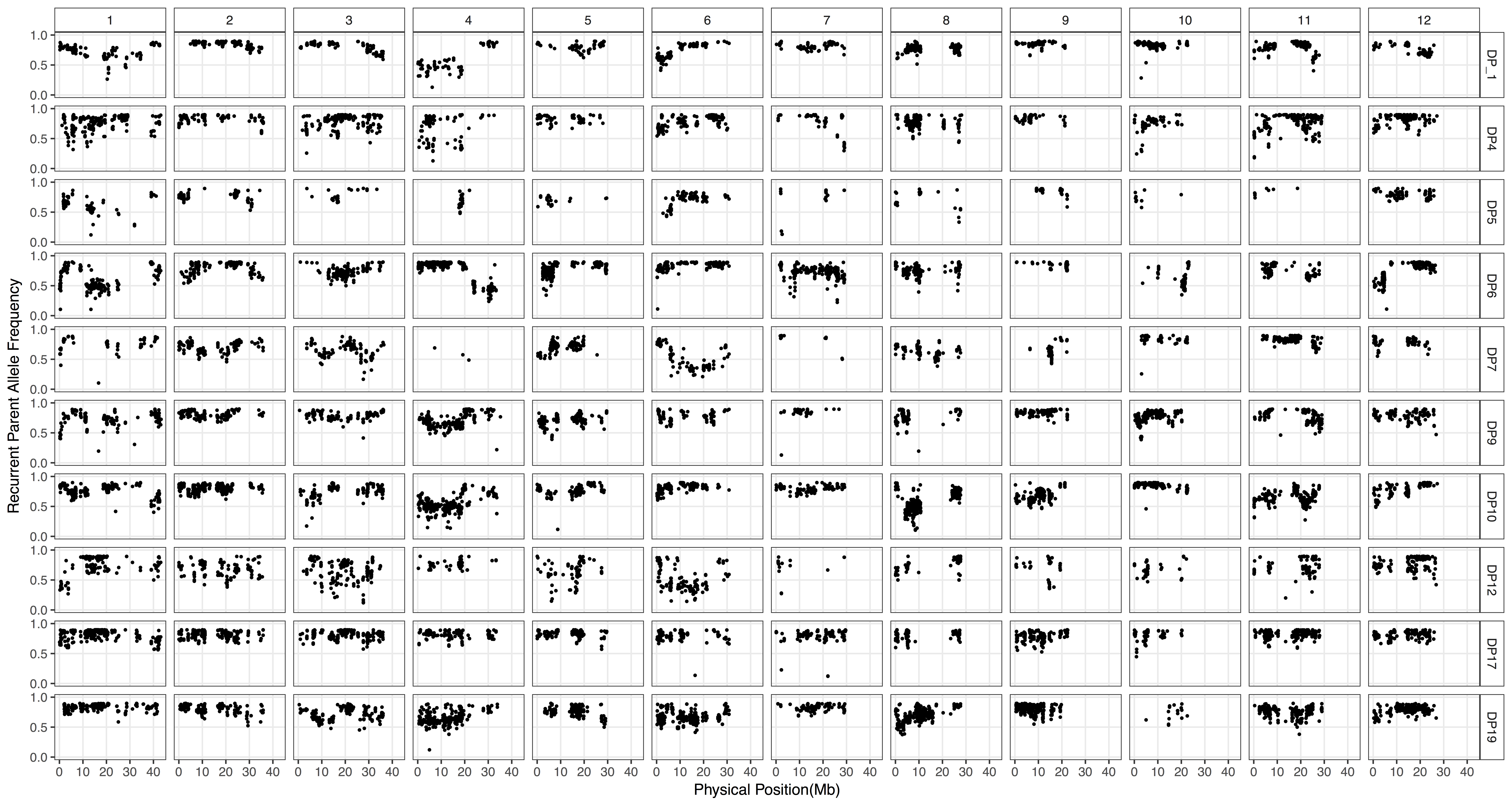

Supplement: FIGURE S10 — Recurrent parent allele frequencies of introgression populations by SNPs (LMD50). Frequency distribution of 12 chromosomes represented for each population titled with its donor parent (DP) identity. [file Image_10.JPEG]

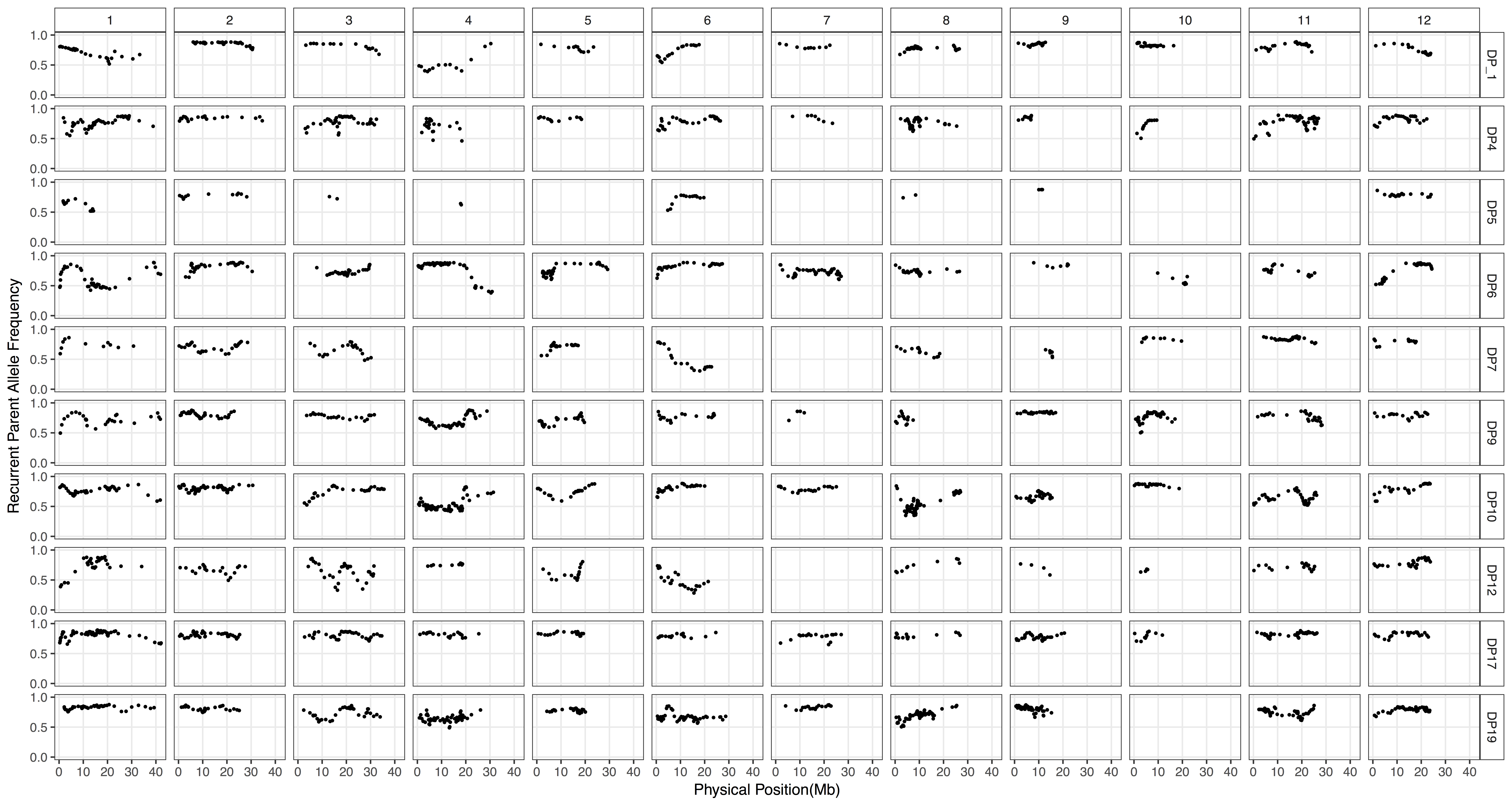

Supplement: FIGURE S11 — Recurrent parent allele frequencies of introgression populations by window scanning method. Frequency distribution of 12 chromosomes represented for each population titled with its donor parent (DP) identity. A window size of 10 SNPs and a step size of 5 SNPs were used. [file Image_11.JPEG]

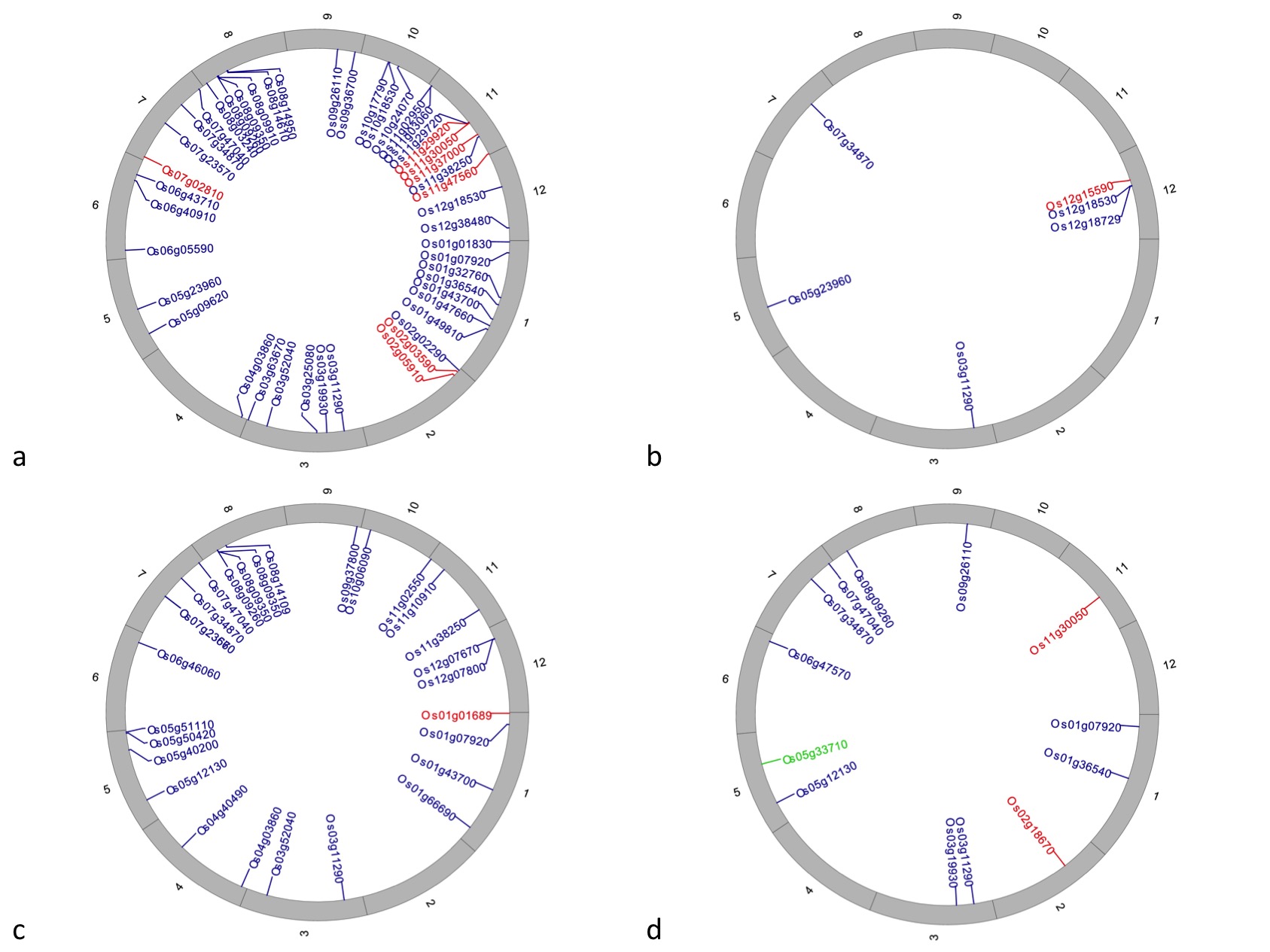

Supplement: FIGURE S12 — Distribution of non-synonymous deleterious SNPs for (a) sub-population 2, (b) sub-population 3, (c) sub-population 4, and (d) sub-population 5. [file Image_12.JPEG]

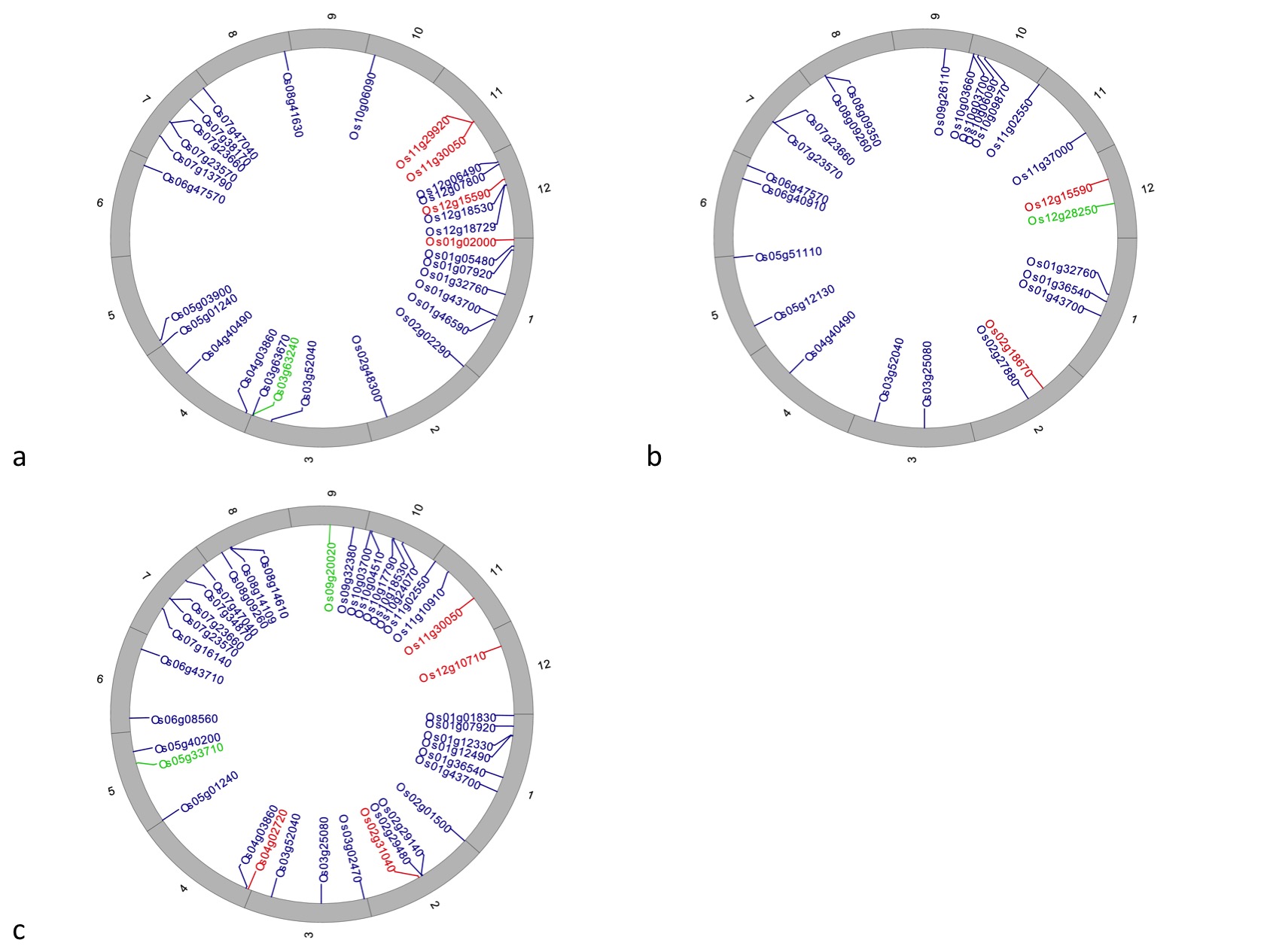

Supplement: FIGURE S13 — Distribution of non-synonymous deleterious SNPs for (a) sub-population 6, (b) sub-population 7, and (c) sub-population 8. [file Image_13.JPEG]
